# Supplementary material for: Etiology of Pneumoparotid: A Systematic Review
Source: J Clin Med. 2022 Dec 24;12(1):144. doi: 10.3390/jcm12010144 (PMC9821654; doi:10.3390/jcm12010144)
Supplement: Supplementary file 1 [file jcm-12-00144-s001.zip › jcm-2044766-supplementary.pdf]

Table S1. Summary of 126 reports on pneumoparotid.

Author	Age	Sex	Side	Duration	Chief complaint	Other symptoms	Past history	Crepitus	Air bubbles	Diagnostic imaging	Findings	Etiology	Group	Treatment	Resolution	Relapse	Follow-up
Deichmüller 1890 [1]	19	m	bi	5 weeks	swelling	-	-	+	NR	-	-	glass blowing	glass blowing	punction	+	-	NR
Scheele 1900 [2]	31	m	bi	NR	recurrent swelling	otalgia, dizziness	epilepsy, measles, scarlet fever	+	+	-	-	glass blowing	glass blowing	NR	NR	NR	NR
	42	m	bi	NR	recurrent swelling	NR	NR	NR	NR	-	-	glass blowing	glass blowing	NR	NR	NR	NR
Scheier 1907 [3]	30	m	bi	15 years	recurrent swelling, pain	pus discharge	larynx catarrh	+	+	-	-	glass blowing	glass blowing	NR	NR	NR	NR
	28	m	bi	13 years	recurrent swelling	-	NR	NR	+	-	-	glass blowing	glass blowing	NR	NR	NR	NR
	21	m	r	NR	recurrent swelling	NR	NR	NR	NR	-	-	glass blowing	glass blowing	NR	NR	NR	NR
	37	m	bi	NR	recurrent swelling	NR	NR	NR	NR	-	-	glass blowing	glass blowing	NR	NR	NR	NR
	39	m	l	5 years	recurrent swelling	pus discharge	NR	+	+	-	-	glass blowing	glass blowing	NR	NR	NR	NR
	37	m	NR	NR	recurrent swelling	NR	NR	NR	NR	-	-	glass blowing	glass blowing	NR	NR	NR	NR
Narath 1912 [4]	42	m	r	8–9 years	recurrent swelling	chronic stomatitis, pharyngitis	arteriosclerosis	+	NR	-	-	glass blowing	glass blowing	rerouting	+	+	several years
Gaus 1941 [5]	23	m	bi	9 years	recurrent swelling	-	mumps	NR	+	radiography	enormous enlargement and part of the Stensen's duct	trumpet	wind instruments	NR	NR	NR	NR
Rysenaer et al. 1963 [6]	13	w	r	NR	recurrent swelling, pain	emphysema in the right face and neck	NR	NR	NR	-	-	nervous tic, self-induced	self-induced	antibiotics	2 weeks	+	NR
	NR	w	bi	NR	swelling, unpleasant tension, pain	NR	mumps	-	-	sialography	normal	nervous tic	diseases	advice not to repeat tic	several days	NR	NR
Rupp 1963 [7]	11	m	bi	short time	recurrent swelling	-	NR	+	+	radiography, sialography	a definite distortion of the arboreal ductal pattern by many cyst-like air spaces	blowing very strongly with the back of the hand occluding the mouth	self-induced	observation	36 hours	+	NR
Reitlinger 1964 [8]	55	m	r	7 years	recurrent swelling, pain	emphysema in the face and neck, sialolith	NR	NR	-	radiography, sialography	dilated duct, air sac	professional trumpet player	wind instruments	antibiotics, surgical incisions, sialolithiasis	+	NR	NR
Greisen 1968 [9]	9	m	bi	6 months	recurrent swelling, pain	emphysema in the face and neck	mumps	+	+	radiography, sialography	air in the soft tissues of the left side of the neck and left parotid region; normal	holding the nose and mouth and then blowing with relaxed, inflated cheeks	self-induced	observation	+	+	18 months
Yoel et al. 1970 [10]	45	m	bi	7–8 months	swelling	nausea, vertigo	NR	NR	-	sialography	normal	siphoner	glass blowing	resigned from the siphoner job	+	-	18 months
	54	m	l	2 months	swelling	-	NR	+	NR	sialography	enlargement of the parotid gland	air bubbles entering when blowing.	glass blowing	sympathetic infiltrations and intraductal instillations	+	-	NR
	57	m	r	NR	swelling, pain	-	NR	NR	+	radiography, sialography	atony of the buccinator muscles and slight inflammation	sneezing crisis	diseases	antibiotics	NR	NR	NR
Rosefsky 1970 [11]	8	m	bi	7 hours	recurrent swelling, pain	NR	mumps, bacterial parotitis	-	-	radiography, sialography	normal	not only dislike of school but also fears about the distortion of and threats to body image	self-induced	psychiatric interviews, Bethanechol	+	-	NR
Calcaterra & Lowe 1973 [12]	14	m	l	1 day	swelling, pain	subcutaneous emphysema over the left parotid gland, pneumomedias tinum	mumps, behavioral problems	+	NR	radiography, fluoroscopy, sialography	subcutaneous emphysema over the left parotid gland	a habit of blowing out the cheeks when upset or disturbed; an adjustment reaction to adolescence	self-induced	antibiotics, psychological counseling	+	+	9 months
Saunders 1973 [13]	NR	NR	NR	NR	recurrent swelling, pain	NR	NR	NR	NR	-	-	trumpet	wind instruments	NR	NR	NR	NR
	NR	NR	NR	NR	recurrent swelling, pain	NR	NR	NR	NR	-	-	clarinet	wind instruments	NR	NR	NR	NR
	NR	NR	NR	NR	recurrent swelling, pain	NR	NR	NR	NR	-	-	wind instrument	wind instruments	NR	NR	NR	NR
	NR	NR	NR	NR	recurrent swelling, pain	NR	NR	NR	NR	-	-	having very forcefully blown up some heavy balloons	balloon	NR	NR	NR	NR

O'Hara & Keohane 1973 [14]	19	m	l	6 months	recurrent swelling, fever	difficulty in breathing	tracheobronchitis, subcutaneous emphysema of the face and neck	+	+	radiography, sialography	a dilated branching ductal structure in the area of the parotid duct; marked dilatation of the parotid duct and evidence of chronic sialadenitis	swelling during sleeping	idiopathic	complete removal of the parotid gland	+	-	NR
Watt 1977 [15]	NR	m	r	NR	emphysema of the right side of the face	NR	xerostomia induced by anti-seasick tablets	NR	NR	sialography	dilated right main duct	inflating the parotid until infection supervened	self-induced	parotidectomy, psychiatric treatment	+	NR	NR
	NR	m	NR	NR	swelling	NR	NR	NR	NR	-	-	decompression after diving	other	NR	NR	NR	NR
	NR	m	NR	NR	recurrent swelling	NR	NR	NR	NR	-	-	watchkeeping in a compartment	other	NR	NR	NR	NR
	NR	m	NR	NR	NR	NR	NR	NR	NR	-	-	bugler (horn)	wind instruments	NR	NR	NR	NR
Sanchez et al. 1980 [16]	15	w	bi	2 years	recurrent swelling, dysphagia	emphysema in the face and neck	personality disorder	NR	NR	sialography	normal; irregularity of the Stensen's duct	self-injury into the Stensen's duct with pins	self-induced	antibiotics, massage	+	NR	NR
Hadas et al. 1982 [17]	16	m	bi	2 years	recurrent swelling, pain	feeling of oppression	NR	NR	+	sialography, US, CT, radiography	air in the parotid glands	mental pathology	diseases	psychiatric counseling	+	NR	6 months
Aristy 1982 [18]	17	m	r	suddenly	swelling	emphysema in the face and neck	asthmatic bronchitis	+	NR	radiography	air in the mandibular angle	asthmatic bronchitis and severe cough crisis	diseases	antibiotics	3 days	NR	NR
Byard 1986 [19]	9	m	L	3 weeks	painful recurrent swelling	-	aphthous ulcer	NR	NR	-	-	a habit of blowing out his left cheek with air for several minutes before meals in an attempt to stop aphthous ulcer pain while eating	abnormal habit	behavioral modification (discouraging cheek inflation)	+	-	6 months
Garber 1987 [20]	32	m	bi	a few minutes	swelling, pain, and pressure when opening jaws	-	hay fever, seasonal allergies	+	-	-	-	forceful clearing of the nares during a hay fever attack	diseases	antibiotics, antihistamine-decongestant	5 days	NR	NR
Markowitz-Spence et al. 1987 [21]	12	w	bi	6 months	recurrent swelling	otalgia, foul taste, erythema of the overlying skin, masseter muscle hypertrophy	chronic obesity, gum chewing, psychosocial adjustment problems at school	NR	-	sialography, CT	air in the duct	Valsalva motion with clenching	self-induced	antibiotics	1 day	+	lost
Komori et al. 1988 [22]	45	m	l	3 years	recurrent swelling	-	diabetes mellitus, otitis media	+	+	US, CT	air in the bilateral glands and ducts	puffing out the cheeks strongly with his breath	abnormal habit	instruction to stop the abnormal habit	-	+	NR
David & Kanga 1988 [23]	6.5	w	l	9 months	swelling	intermittently warm, erythematous, tender	cystic fibrosis	+	+	radiography, CT	loculated cystic dilation with several rounded gaseous latencies	habitually filling the left cheek with air and pressing on it forcefully with the fist, attempting to suppress frequent cough	abnormal habit	antibiotics, percutaneous evacuation, reinforcement to abolish the tic	+	-	NR
Brodie & Chole 1988 [24]	14	m	r	1 year	recurrent swelling	subcutaneous emphysema in the right face	left maxillary fracture with massive subcutaneous emphysema on both sides of the face and neck	+	+	CT	the air throughout the duct and swelling of the gland with air	causing swelling by increasing intraoral pressure	self-induced	rerouting the parotid duct to the apex of the tonsillar fossa	NR	-	3 years
Telfer & Irvine 1989 [25]	29	m	r	2 years	squelching noise and feeling	-	-	+	+	radiography, US, sialography	dilating ducts containing air	idiopathic	idiopathic	lengthening and posteriorly repositioning of the duct by using a buried mucosal flap based on a periductal pedicle	+	-	4 months
Yamashita et al. 1990 [26]	40	m	r	suddenly	swelling	-	-	NR	NR	CT	air density area in both parotid gland	massage in the periauricular region	other	punction guided with US	immediately	-	NR
Mandel et al. 1991 [27]	53	m	r	3 years	recurrent swelling, discomfort	tenderness	-	+	+	sialography	contaminated ductal air causes a widely dilated primary duct with evidence of spherical radiolucency	a habit of blowing out the cheek	abnormal habit	advice	NR	NR	NR

Piette & Walker 1991 [28]	34	w	r	suddenly	swelling, sharp pain	-	NR	+	NR	radiography	well-demarcated radiolucent area	air syringe used during dental treatment	iatrogenic	antibiotics	+	NR	NR
Takenoshita et al. 1991 [29]	24	m	l	immediately	"squelching" or "squeaking noise," swelling	painful sensation	-	+	-	US	air in the left parotid gland as well as in the subcutaneous tissue	flashing the air syringe between the tray with hardened alginate impression	iatrogenic	antibiotics	2 days	-	1 year
Krief et al. 1992 [30]	10	m	bi	a few days	swelling, pain	NR	NR	+	-	CT, sialography	extensive cystic air collections mainly in the right parotid, air throughout both parotid ducts and local buccal air pockets near the orifices of Stensen's ducts; normal ducts bilaterally with spillage of contrast into the air cysts on the right	a habit of forcefully blowing out the cheeks	self-induced	antibiotics, advice, training	-	+	lost
Curtin et al. 1992 [31]	36	m	bi	9 years	recurrent swelling	-	NR	+	NR	sialography, radiography, US	sialectatic air-containing ducts; multiple small hyperechoic areas bilaterally corresponding to air within the ducts	performing a Valsalva maneuver with an open glottis and nose occluded in an attempt to clear the ears	abnormal habit	advice not to puff out the cheeks	+	NR	NR
Ferlito et al. 1992 [32]	14	m	bi	2 years	recurrent swelling, pain	-	nervous tic	NR	-	US, sialography, radiography	US: enlarged right parotid gland with various hyperechoic areas and acoustic shadows due to the presence of gas, sialography; no abnormalities, radiography: the presence of gas bubbles within the right parotid	nervous tic consisting of his puffing out the cheeks with closed lips	diseases	antibiotics, anti-inflammatory drugs, corticosteroids	NR	NR	NR
Brown et al. 1992 [33]	30	m	l	shortly	shooting pain, swellin	-	-	-	-	-	-	barotrauma to the parotid secondarily the use of an air-powder prophylaxis unit	iatrogenic	NSAIDs, antibiotics	5 days	NR	NR
Terahara et al. 1992 [34]	9	m	bi	2 years	recurrent swelling	emphysema in the face and neck	mumps	+	NR	radiography, CT	air in both glands; emphysema in the face and neck	blowing the cheeks	self-induced	advice not to blow the cheeks	1 day	+	NR
Yonetsu et al. 1993 [35]	64	m	l	5–6 years	swelling	-	NR	NR	+	radiography, CT	loculated gaseous in the parotid region, the air in Stensen's duct, and the parotid gland ducts	idiopathic	idiopathic	refused	NR	NR	NR
Birzgalis et al. 1993 [36]	16	m	r	2 months	pain, swelling	subcutaneous emphysema of the face and neck	pleomorphic adenoma	NR	-	CT	a rounded cystic lesion within the gland	elevating skin flap at will by a Valsalva maneuver	self-induced	antibiotics, advice	+	NR	NR
McDuffie et al. 1993 [37]	24	m	bi	5 days	swelling	-	-	-	-	-	-	puffing cheeks to relieve irritation from the orthodontic braces	abnormal habit	instruction to refrain from puffing the cheeks	2 days	NR	NR
Cook & Layton 1993 [38]	44	w	bi	NR	chronic swelling	-	congenital kyphoscoliosis, chronic obstructive pulmonary disease	-	-	radiography, sialography	normal	increased intraoral pressure needed for respiration during coughing attacks	diseases	NR	NR	NR	NR
Nassimbeni et al. 1995 [39]	12	m	bi	1 year	recurrent swelling	subcutaneous emphysema	anxiety attacks and headaches	+	NR	sialography, CT	confirmation of clinical picture	blowing it up and deflating it	self-induced	extensive treatment with a child psychiatrist and psychologist	+	-	8 years
	9	m	r	NR	recurrent swelling	pyopneumoparotitis	unhappy family situation	NR	NR	radiography, US, sialography	presence of air bubbles	inflating and deflating the parotid at will	self-induced	aspiration, antibiotics, surgical incisions	+	-	NR
Goguen et al. 1995 [40]	9	m	r	3 days	swelling	-	NR	NR	+	CT	air in the right parotid gland and duct	blowing hard against the palm	abnormal habit	antibiotics, explanation of the maladaptive behavior, and potential complications	1 day	-	NR
	9	w	bi	a long history	recurrent swelling	tenderness	recurrent parotitis	+	-	CT	air within the left and right glands	a habit of repeatedly blowing up the parotid gland by Valsalva's maneuver with a closed mouth	abnormal habit	antibiotics, advice to stop the behavior, left superficial parotidectomy	NR	-	NR

	13	m	bi	5 years	recurrent swelling	-	recurrent juvenile parotitis	NR	+	radiography, sialography	radiography: air in the parotid region, sialography: normal	blowing up his cheeks, which always occurred on Sunday afternoons and possibly a maneuver to avoid school	self-induced	psychiatric counseling	NR	NR	NR
Nakamura et al. 1996 [41]	10	m	bi	4 years	recurrent swelling, pain	emphysema in the temporal region	acute parotitis, acute otitis media, acute sinusitis, cervical lymphadenitis	+	-	radiography, CT	air in the right gland, emphysema in the temporal region	self-inflating the parotid glands, psychological problems	self-induced	antibiotics, advice not to blow his cheeks	2 weeks	+	NR
Ros et al. 1996 [42]	3	m	l	1 day	swelling, pain	-	-	-	-	-	-	blowing balloon	balloon	consumption of orange juice, which resulted in the expulsion of the trapped air	immediately	NR	NR
Eligi et al. 1997 [43]	50	m	l	2 months	recurrent swelling, pain	NR	-	NR	-	radiography, US, CT	air in the enlarged duct and gland	idiopathic	idiopathic	antibiotics	+	-	6 months
Barthold 1998 [44]	18	w	bi	5 years	recurrent swelling	subcutaneous emphysema of the neck	tonsillectomy	+	+	US, sialography, CT, MRI	air in the bilateral parotid glands	Valsalva's maneuver	abnormal habit	antibiotics	NR	NR	NR
Gudlaugsson et al. 1998 [45]	16	w	bi	9 months	recurrent swelling, tenderness	subcutaneous emphysema, pneumomediastinum	orofacial tics	+	NR	CT	parotid swelling, air both within the parotid glands on the right side, air in the soft tissue extending from the parotid region down the neck to the thyroid gland	a habit of biting the lower lip and whistling with a high-frequency sound	abnormal habit	explanation of the risk of the habit	within 24 hours	twice	1 year
Alcalde et al. 1998 [46]	29	m	r	3 months	pain, swelling	bilateral tinnitus, headache	trauma to the head and maxillofacial region	+	+	CT, sialography	a round, well-circumscribed radiolucency compatible with air within the parotid gland air within the parotid gland	trauma to the head and maxillofacial region	diseases	needle aspiration, NSAIDs, antibiotics	NR	NR	refused
Leuwer & Greess 1998 [47]	25	m	l	3 months	recurrent swelling	-	NR	+	NR	CT	the air-filled parotid duct on the left, bubble-like accumulations of air appeared in the parotid	cheek puffing	abnormal habit	NR	NR	NR	NR
Golz et al. 1999 [48]	10	m	bi	2 years	recurrent swelling	-	mumps	-	-	CT	bilateral enlargement of the parotid glands, extensive cystic air collections in the parotid glands' parenchyma, and air throughout both parotid ducts	holding the nose and mouth and blowing out the cheeks whenever he did not want to go to school	self-induced	psychological evaluation and counseling	+	-	1 year
Sittel et al. 1999 [49]	14	w	bi	3 years	recurrent swelling	-	constant itching in both external ear canals	NR	+	MRI	massive luminal widening of Stensen's duct, complete loss of signal inside the parotid duct due to air insufflation	puffing the cheeks whenever the itching became unbearable	abnormal habit	antibiotics, advice to change the behavior	few days	NR	lost
Kirsch et al. 1999 [50]	41	m	l	immediately	swelling	sense of fullness without pain	viral upper respiratory tract infection	-	-	CT	air within the left parotid gland and Stensen's duct.	spirometry; sometimes producing facial swelling at will by coughing or blowing forcefully against the closed mouth	iatrogenic	NR	NR	NR	NR
Martín-Granizo et al. 1999 [51]	5	w	bi	NR	pain, recurrent swelling	-	recurrent parotitis and sialectasis	NR	NR	radiography, US, CT	an increased density of the left parotid parenchyma with multiple air acinic images	idiopathic	idiopathic	antibiotics, hydration, warm compresses	NR	NR	NR
	8	w	r	1 week	pain, recurrent swelling	-	febrile convulsions, bronchitis with intense spasms of coughing	+	NR	sialography, CT	several air bubbles inside the parotid parenchyma	excessive intraoral pressure provoked by repeated spasms of coughing	diseases	NR	NR	NR	NR
Huang et al. 2000 [52]	50	m	bi	a long history	nasal congestion	-	postnasal drainage, nasal congestion	NR	-	CT	air in the bilateral Stensen's ducts	idiopathic	idiopathic	observation	NR	-	1 month
Yokoyama et al. 2001 [53]	9	m	bi	3 months	recurrent swelling, pain	subcutaneous emphysema in the left face and neck	mumps	+	+	CT	air in the left parotid gland, face, and neck	idiopathic	idiopathic	antibiotics	6 days	+	6 months
Cho et al. 2001 [54]	42	m	l	NR	swelling	-	-	NR	+	CT, sialography	small air collection in the left parotid gland	trumpet playing with puffed cheeks	wind instruments	advice not to play trumpet with puffed cheeks	NR	NR	NR
Franco et al. 2002 [55]	10	w	l	several days	swelling	subcutaneous emphysema in the left neck	mumps	NR	-	CT	air in the left parotid gland and duct	nervous tic; autoinsufflation	diseases	etiologic and symptomatic treatment	NR	NR	NR

Brasseur et al. 2003 [56]	31	w	bi	NR	recurrent swelling	dysphagia, cervical and facial subcutaneous emphysema	asthma, borderline personality disorder, Munchausen syndrome	+	+	US, CT	numerous intraparenchymal hyperechogenic islets corresponding to air bubbles	auto-mutilation and intentional production of physical symptoms	self-induced	antibiotics, neuroleptic, antidepressant, anxiolytic	15 days	NR	lost
Orabi & Nigam 2004 [57]	21	w	bi	13 years	recurrent swelling, pain	subcutaneous emphysema	asthma, miscarriage	-	-	radiography, sialography, CT, US	air in both parotids as well as in subcutaneous tissue	self-induced	self-induced	psychological counseling	1–2 days	+	lost
Han and Isaacson 2004 [58]	13	m	r	NR	recurrent swelling	subcutaneous emphysema in the face and neck	left recurrent parotitis, attention deficit hyperactivity disorder	+	NR	sialography, CT	extensive air in the ductal system with extension into the soft tissues from the skull base to the lung apex	idiopathic	idiopathic	antibiotics, ductal ligation	1 week	-	2 years
Apaydin et al. 2004 [59]	50	m	l	18 months	recurrent swelling	tenderness, xerostomia	-	+	-	CT, MRI	a big cyst containing air in the left parotid gland	idiopathic	idiopathic	cystectomy	+	3 days after	6 months
Maehara et al. 2005 [60]	15	m	bi	a long history	recurrent swelling, tenderness	-	mumps	NR	NR	CT	very small amounts of air throughout most of the right parotid	a habit of blowing out the cheeks	abnormal habit	explanation and advice to not blow the cheeks	+	NR	NR
De Meerleer & Hermans 2005 [61]	20	w	bi	1 year	recurrent painful swelling	NR	NR	+	+	CT	bilateral enlargement of the parotid glands, with several intraglandular air collections (more on the left side), as well as air-filled dilation of Stensen's duct on the left side	idiopathic	idiopathic	surgical repositioning of both parotid ducts' orifices via a submucosal tunnel (Wilkie-Brody procedure)	NR	NR	NR
Grainger et al. 2005 [62]	12	w	bi	6 months	recurrent swelling, pain	NR	NR	NR	NR	sialography, CT	mild chronic inflammation; air in both parotid glands	idiopathic	idiopathic	conservative management (antibiotics and analgesia)	several hours and days	+	9 years
Paskoy et al. 2006 [63]	38	m	bi	several years	swelling	NR	NR	NR	+	CT, US	air within bilateral parotid glands	induced by the Valsalva technique	abnormal habit	Advice	+	-	1 year
Scherr et al. 2006 [64]	34	m	r	a few hours	discomfort, crepitus	NR	NR	+	NR	US, CT	an air-filled Stensen's duct, which is thickened when compared to the sides, and predominantly blister-like, partly also longitudinally arranged accumulations of air in the entire parotid parenchyma	very forced and recurrent increase in intraoral pressure by blowing into the fanfare	wind instruments	antibiotics	1 week	-	NR
Adachi 2006 [65]	59	m	r	suddenly	swelling	emphysema in the face and neck	purulent meningitis, mental retardation	+	NR	radiography, CT	air in the parotid gland and enlargement of the duct	a habit of repeatedly blowing out the mouth	abnormal habit	antibiotics	-	+	NR
Yang et al. 2007 [66]	10	w	r	1 day	swelling	subcutaneous emphysema in the neck and mediastinum	NR	+	NR	CT, US	air in bilateral parotid glands and ducts	playing flute	wind instruments	antibiotics, needle aspiration	7 days	-	NR
Chun et al. 2007 [67]	18	m	bi	3 months	swelling, pain	NR	NR	+	+	CT	air within bilateral parotid glands	insufflation of air within the gland with a closed mouth to take a break from work	self-induced	antibiotics, cognitive behavioral therapy	5 days	-	7 months
Balasubramanian et al. 2008 [68]	11	m	bi	2 years	recurrent swelling	pus discharge from the duct, enlargement of the left parotid gland, subcutaneous emphysema	-	+	-	sialography, CT	evidence of reduced function in the left parotid gland; left-sided pneumoparotid	with a handkerchief in hand and blow forcefully into the closed hand puffing out the cheeks like a Valsalva-like maneuver	self-induced	counseling	dramatic recovery	-	NR
Núñez et al. 2008 [69]	28	w	l	NR	swelling	cracking on palpation	Down's syndrome	+	NR	radiography, CT	a large amount of air was identified in the parenchyma and the internal duct system of the parotid gland	insufflation of air within the gland by performing forced Valsalva techniques to obtain the attention and care of parents	self-induced	NR	+	-	NR
Luaces et al. 2008 [70]	11	m	r	15 days	pain, swelling	subcutaneous emphysema	-	+	+	CT	dilated right intraglandular and extra glandular ducts and also a moderate amount of air in the right parotid duct	the unconscious habit of blowing out the cheeks as an	abnormal habit	antibiotics, massage	NR	-	1 year

Joo et al. 2008 [71]	20	m	l	1 month	swelling	bilateral hearing impairment	NR	+	+	CT	enlargement of the left duct and air in the duct	lifting of heavy luggage	other	antibiotics, massage, ligation of left Stensen's duct	2 weeks	-	1 year
Prabhu & Tran 2008 [72]	12	m	bi	NR	recurrent swelling	-	NR	NR	NR	CT	extensive ductal and acinar pneumatic dilation; air in both parotid glands and Stensen's ducts	puffing out of the cheeks to prevent irritation by orthodontic braces	abnormal habit	antibiotics, counseling	NR	NR	NR
Faure et al. 2009 [73]	9	m	l	3 years	recurrent swelling	emphysema extending into the left cheek, the parotid gland region, and the adjacent lateral cervical region	NR	+	+	CT	air inside the left parotid duct, as well as extra ductal air within the parotid gland, subcutaneous soft tissues, and even in the parapharyngeal space	puffing the cheeks in stressful situations; conflicts with the child's parents as the most probable cause of chronic stress	abnormal habit	antibiotics, psychiatric counseling	NR	NR	NR
Mukundan & Jenkins 2009 [74]	13	m	l	2 days	swelling, pain	-	NR	NR	+	CT	air in the left parotid gland and the parotid duct	playing tuba	wind instruments	conservative management	within days	NR	NR
Lee et al. 2009 [75]	21	m	l	6 months	swelling	-	NR	+	+	CT	air in the left duct and the gland	a habit of blowing the cheeks	abnormal habit	instruction to avoid blowing cheeks	+	1 year	NR
Moënne et al. 2009 [76]	13	m	r	3 hours	swelling	tenderness, swelling	NR	NR	NR	US	thickness of the parotid parenchyma, presence of echogenic linear images with the appearance of corresponding to air, which also demonstrated throughout the length of Stensen's duct	inflating balloons for hours	balloon	analgesia	2 days	-	3 years
	10	m	r	NR	pain, swelling	-	NR	NR	NR	US	presence of a small amount of air in the intraglandular ducts, in both parotids, without other associated alterations	inflating lots of balloons	balloon	analgesia	2 days	-	NR
	5	w	l	several hours	pain, swelling	tenderness	NR	NR	NR	US	repeatedly submerging in the pool to make "bubbles," for what which she kept the air in the oral cavity before getting the water	repeatedly submerging in the pool to make "bubbles," for what which she kept the air in the oral cavity before getting the water	other	NR	+	-	NR
	9	w	r	2 days	pain, swelling	-	NR	NR	NR	US	increased volume of the parotid right, with focal areas of increased echogenicity, in the thickness of the gland, a linear path echogenic suggestive of corresponding to air	blowing a hard wooden flute	wind instruments	NR	rapidly	-	NR
Kyung et al. 2010 [77]	7	w	bi	NR	pain, extensive edema of the face and neck	subcutaneous facial and cervical emphysema	-	NR	NR	CT	presence of numerous air bubbles in both parotids and Stensen's duct, with gaseous effusion extending to the neck and mediastinum	a very energetic and unusual way of blowing on a new recorder with all holes covered	wind instruments	putting a stop to the trigger factor, antibiotics, and analgesics	a few days	1 month later	2 years
Fukuta et al. 2011 [78]	34	m	r	6 months	recurrent swelling, discomfort	-	-	-	+	CT, MRI	air in both parotid glands, enlargement of both ducts	a habit of blowing out the cheeks	abnormal habit	advice not to blow the cheeks	+	NR	8 months
Kolti 2011 [79]	27	m	l	NR	recurrent pain, swelling	-	NR	+	NR	-	-	inflating toys and balloons	balloon	instruction to avoid the precipitating factor (balloon-blowing)	NR	NR	NR
van Ardenne et al. 2011 [80]	7	w	l	1 month	swelling	-	NR	-	-	CT	bilateral dilated intraglandular and extra glandular ducts, a large amount of air in the left parotid gland, and a small amount of air in the right parotid gland and duct	a habit of blowing out her cheeks in a stressful situation	abnormal habit	explanation and advice not to blow up the cheeks	NR	NR	NR
Iwaki et al. 2011 [81]	20	m	r	NR	swelling	-	NR	+	+	CT	air in the parotid gland, enlargement of Stensen's duct	a habit of blowing the cheeks	abnormal habit	instruction and massage	1 week	NR	NR
Vasi & Hoskins 2011 [82]	30	m	bi	22 years	recurrent pain and swelling	sound of "wind"	-	+	+	CT	air within ducts and parotid glands	idiopathic	idiopathic	antibiotics	-	+	NR
Zuchi et al. 2011 [83]	50	w	l	3 years	pain, swelling	edema in the left cervical and mandibular regions	NR	NR	NR	US, CT	US: a solid nodule with small peripheral calcification on the left gland, CT: multiple air-filled small	idiopathic	idiopathic	sulphamethoxazole, trimethoprim, local	-	+	1 year

											cavitations within the ductal tree and in the Stensen duct			symptomatic measures			
Ghanem et al. 2012 [84]	46	m	l	5 years	discomfort, swelling	-	NR	+	+	US, sialography	multiple hyperechoic spots throughout the gland, representing small focal collections of air	idiopathic	idiopathic	NR	NR	NR	NR
Tekelioglu et al. 2012 [85]	57	w	l	after general anesthesia	swelling	-	hypertension	+	NR	-	-	general anesthesia	iatrogenic	observation	2 days	-	NR
Tachibana et al. 2012 [86]	7	m	l	1 month	recurrent swelling, tenderness	subcutaneous emphysema	asthma	+	NR	CT	air in the left parotid, expanding subcutaneous emphysema	"inflatable" mouth	abnormal habit	antibiotics, advice not to inflate the mouth	2 days	-	3 months
Li et al. 2012 [87]	18	m	l	6 months	swelling	facial trauma	NR	NR	+	CT, sialendoscopy	gas in the left parotid gland, bubbles in the Stensen's duct, enlargement of the duct	facial trauma	other	antibiotics	-	-	6 months
McCormick et al. 2013 [88]	7	m	bi	4 years	recurrent swelling	tenderness	G6PD deficiency, mild obstructive sleep apnea syndrome, allergic rhinitis	NR	NR	CT	cystic air-filled dilations were identified within the parotid glandular tissue	idiopathic	idiopathic	antibiotics, NSAID	NR	NR	NR
Potet et al. 2013 [89]	44	w	l	2 weeks	swelling, pain	slight fever	NR	NR	NR	US, MRI, CT	air within the left parotid duct and left parotid parenchyma.	idiopathic	idiopathic	antibiotics	+	NR	NR
McGreevy et al. 2013 [90]	48	m	bi	25 years	recurrent swelling, pain	infection of the gland	-	+	+	CT	air within both parotid glands, a fluid level with cystic changes, and dilated air-filled intraparotid ducts in the right gland	self-insufflating both parotid glands by expiring against a closed mouth	self-induced	right parotidectomy	NR	NR	NR
Pillay & Goh 2014 [91]	61	w	r	immediately	swelling	-	diabetes mellitus, hypertension, systemic lupus erythematosus	-	NR	-	-	upper endoscopy	iatrogenic	observation	3 hours	-	1 week
Watanabe et al. 2014 [92]	52	m	r	suddenly	pain, swelling	-	gout, hyperlipidemia	-	+	CT	air in the right parotid gland	air syringe used during dental treatment	iatrogenic	antibiotics	NR	-	1 month
Nicot et al. 2014 [93]	8	m	r	2 years	recurrent swelling	-	NR	NR	-	CT	air in the gland and duct	dive holding breath	other	change in habits (stopping the dive holding breath during swimming sessions)	2–3 days	NR	NR
Almario Hernández et al. 2014 [94]	7	m	L	soon	swelling	-	NR	+	NR	US	normal	blowing balloon	balloon	antibiotics	+	NR	NR
Konstantinidis et al. 2014 [95]	61	m	bi	2 years	recurrent swelling	-	right sialadenitis	-	+	CT	the presence of air in both ductal systems with more prominent in the right parotid gland	idiopathic	idiopathic	sialendoscopy and irrigation with steroids	6 months	twice	6 months
Ino et al. 2015 [96]	40	m	bi	1 week	pain, swelling	fever, cough	mumps	-	+	CT	air in the bilateral ducts and parotid glands	elevation of intraoral pressure due to suppression of cough	diseases	antibiotics	10 days	+	NR
	61	w	r	10 years	recurrent swelling	-	xerostomia	-	-	CT	air in the right enlarged duct	blowing mouth to relieve discomfort	abnormal habit	minor tranquilizer, advice	NR	NR	6 months
	24	m	R	2 years	swelling	-	recurrent parotitis	-	-	CT	air in the right duct	puffing out the cheeks	abnormal habit	antibiotics, minor tranquilizer, advice	NR	NR	lost
	15	m	bi	NR	recurrent swelling	NR	NR	NR	NR	CT	air in the left gland	swelling before the exam or after parents' blame	self-induced	NR	NR	NR	NR
	52	w	r	NR	NR	NR	insomnia	NR	NR	CT	air in the right gland	puffing out the cheeks in a nervous or uncomfortable situation	abnormal habit	NR	NR	NR	NR
	33	w	r	NR	feeling of fullness	NR	NR	NR	NR	CT	air in the right gland	puffing out the cheeks	abnormal habit	NR	NR	NR	NR

	56	m	l	NR	NR	NR	stress	NR	NR	CT	air in the left gland	blowing mouth when tired	abnormal habit	NR	NR	NR	NR
	17	m	r	NR	NR	NR	stress	NR	NR	CT	air in the right gland	blowing mouth in a stressful situation	abnormal habit	NR	NR	NR	NR
Dietrich et al. 2015 [97]	33	m	r	NR	swelling	NR	NR	+	-	US, sialography, CT	air in the right duct	blowing balloon	balloon	instruction to avoid the precipitating factor (balloon-blowing)	NR	NR	NR
Bowden & Bowden 2015 [98]	4	m	l	soon	face and neck swelling, pain	cervicofacial subcutaneous emphysema	NR	+	NR	radiography	subcutaneous emphysema in the left paramandibular area and left-sided neck	blowing balloon	balloon	instruction to refrain from performing any activities that can cause high intraoral pressure, such as blowing up a balloon	3–4 days	NR	NR
Cabello et al. 2015 [99]	42	m	r	3 months	swelling, pain	-	moderate obstructive sleep apnea syndrome	+	NR	US, CT	normal morphology; the presence of numerous air bubbles in the right parotid and gas along the right duct	blowing all night, and sometimes his wife heard an intense whistle.	diseases	NSAIDs	NR	NR	NR
Osawa et al. 2015 [100]	11	m	l	2 days	recurrent swelling, pain	emphysema in the face and neck; pneumomediastinum	mumps, cyclic vomiting syndrome	+	-	CT	air in bilateral glands and neck	elevation in oral pressure due to suppression of vomiting	diseases	antibiotics, advice not to blow the cheeks due to suppression of vomiting	6 days	+	NR
Shibata & Harada 2016 [101]	6	m	l	1 month	recurrent swelling, pain	-	mumps	NR	+	CT	air in the Stensen's duct	a habit of blowing the cheeks	abnormal habit	antibiotics, instruction to stop blowing cheeks	1 month	-	NR
	43	w	l	6 days	pain, swelling	-	-	NR	-	CT	enlargement of Stensen's duct due to the retrograde passage of air and emphysema in the left parotid gland	idiopathic	idiopathic	antibiotics	1 month	-	NR
Abdullayev et al. 2016 [102]	36	NR	bi	3 days	swelling	chronic obstructive pulmonary disease	congenital bronchiectasis, chronic obstructive pulmonary disease	NR	NR	US	parotitis	non-invasive positive pressure ventilation	iatrogenic	antibiotherapy, non-invasive CPAP, bronchodilator therapy	NR	NR	NR
Lagunas & Fuertes 2017 [103]	13	m	bi	5 years	recurrent swelling	emphysema in the neck and face	recurrent chronic parotitis	NR	NR	CT	presence of air inside the entire path of the duct extending into the parotid gland and dissecting through the deep space of the neck into the parapharyngeal space and the infratemporal fossa	aspirating deeply inside the mouth	self-induced	antibiotics, NSAIDs	NR	NR	NR
Alnæs & Furevik 2017 [104]	primary school age	w	l	a few days	swelling, pain, redness of the skin	emphysema in the left face	-	NR	NR	US, CT	artifacts consistent with air; pronounced emphysema throughout the left side of the face	blowing on a paper trumpet and sucking on water bottles	wind instruments	antibiotics, aspiration of the parotid duct	rapidly	+	NR
Oh et al. 2017 [105]	21	w	bi	3 weeks	swelling, pain	-	NR	+	+	CT	multiple-filled spaces within the bilateral parotid glands and air in bilateral dilated Stensen's ducts	a habit of blowing the cheeks	abnormal habit	instruction not to blow the cheeks	1 week	-	1.5 years
Lee et al. 2017 [106]	11	m	l	6 hours	swelling, pain	emphysema in the neck and face, pneumomediastinum	-	+	NR	radiography, US, CT	extensive subcutaneous emphysema involving the left side of the face and neck	idiopathic	idiopathic	antibiotics	5 days	NR	NR
Kwon et al. 2017 [107]	38	w	l	3 years	recurrent swelling	-	masseter pain	+	+	CT	air in the left parotid gland	a habit of blowing out the cheeks	abnormal habit	instruction to avoid blowing out the cheeks	+	-	5 months
Goates et al. 2018 [108]	53	m	l	2 months	swelling	foul-tasting, thick mucus draining	obstructive sleep apnea syndrome,	NR	+	CT, sialendoscopy	air in the left parotid gland	CPAP for 5 years	iatrogenic	transfer from full-face to nasal CPAP	2 weeks	-	6 months

	54	m	r	7 months	recurrent swelling	parotitis	Sjögren syndrome, Warthin's tumor obstructive sleep apnea syndrome, Sjögren syndrome, Warthin's tumor	NR	NR	sialography	parotid gland strictures with multiple air locules with distal megadilation of the duct	CPAP for 10 years	iatrogenic	antibiotics, transfer from full-face to nasal CPAP	-	+	1 year
Yamazaki et al. 2018 [109]	53	m	bi	1 year	"squishy" sound	-	vitiligo vulgaris	+	+	radiography, US, MRI	enlargement of the left parotid duct and high-echogenicity areas inside the bilateral parotid ducts	bilateral compression of the buccal region	abnormal habit	instruction to stop compressing the buccal region	+	+	NR
Paterson et al. 2018 [110]	24	m	l	6 months	swelling	-	NR	NR	NR	CT	a 4.5 cm by 3.5 cm air pocket within the left parotid gland	idiopathic	idiopathic	needle aspiration	+	NR	NR
House & Lewis 2018 [111]	34	m	bi	NR	recurrent sensation of a "pop"	emphysema in the head and neck, nausea, vomiting	bipolar disorder	+	NR	CT	subcutaneous emphysema of the left facial and bilateral neck soft tissues, from the level of the scalp to the sternal notch	self-induced to leave prison	self-induced	antibiotics	-	+	NR
Miłoński et al. 2019 [112]	54	m	l	3 weeks	swelling	pain	NR	NR	NR	US, CT	a thickening of the parotid parenchyma with a low-echoic focal lesion; air in the excretory ducts, and an air bubble about 2 cm in diameter in the vicinity of the gland	a habit of putting the tongue into the left cheek	abnormal habit	conservative treatment (a pressure dressing), excision of the left pneumoparotid	+	NR	NR
Ambrosino et al. 2019 [113]	12	m	bi	2 years	recurrent swelling, pain	subcutaneous emphysema	juvenile Sjögren syndrome	+	+	CT, sialendoscopy	uncommon aeric formation	idiopathic	idiopathic	antibiotherapy, parotid massage, painkillers	-	+	NR
Basha 2019 [114]	40	m	r	1 year	recurrent swelling	tenderness	-	NR	+	US, CT	mild enlargement and a hyperechoic cluster of foci with posterior acoustic shadow likely of being a calculus; right parotid enlargement with multiple free air foci within the gland parenchyma, discrete dense foci of small calcifications	insufflation of air from the mouth	abnormal habit	advice not to hold air mouth and blow with pressure	+	-	6 months
Raczkowska -Łabuda et al. 2019 [115]	12	m	bi	6 months	swelling	-	NR	NR	NR	CT, US, sialography	a 23 mm space filled with air in the right parotid region, air in the salivary ducts of the right parotid gland	deliberate inflating of both parotid glands	self-induced	antibiotics, irrigation	+	-	3 months
Kim et al. 2019 [116]	67	m	l	NR	parotid mass	-	NR	NR	NR	CT	a 1 cm-sized enhancing nodular lesion in the left parotid gland	Idiopathic	idiopathic	observation	-	-	4 years
Enami et al. 2020 [117]	37	w	l	suddenly	swelling, pain	-	-	-	-	CT	air in the left parotid gland	dental examination using an air syringe	iatrogenic	antibiotics	2 weeks	-	NR
Yang & Bundrick 2020 [118]	30s	m	bi	NR	pain, swelling	subcutaneous emphysema in the neck and mediastinum	-	NR	+	CT	intraluminal air in the left parotid duct with associated pneumoparotid and dissection of air into the left parapharyngeal and masticator spaces	puffing out the cheeks to force air into the neck with subsequent decompression of air back into the oral cavity by pushing behind the ear	abnormal habit	antibiotics	NR	NR	NR
Sinha et al. 2020 [119]	50	w	bi	NR	recurrent swelling	NR	-	+	+	US	presence of hyperechoic air bubbles within the duct and the surrounding salivary gland tissue	habitually puffing the cheeks	abnormal habit	encouragement to break the habit	NR	NR	NR
Gazia et al. 2020 [120]	45	m	l	NR	painful swelling	subcutaneous emphysema that affected caudo-cranial left soft tissues from the temporal region to the upper thoracic outlet	cognitive disability	+	NR	CT	air in the left parotid gland, severe ectasia of Stensen's duct	idiopathic	idiopathic	antibiotics, psychiatric therapy	NR	NR	NR
Gray et al. 2020 [121]	9	w	l	1 month	swelling	subcutaneous emphysema	trisomy 21 (Down's syndrome)	NR	NR	CT	gas within the ductal system of the parotid gland	voluntarily blowing up her cheeks	abnormal habit	fine needle aspiration	NR	NR	NR

Aljeaid et al. 2020 [122]	12	m	l	3 months	recurrent painless swelling	temporal subcutaneous emphysema	-	NR	-	CT	presence of a rounded air-filled left parotid, bilateral dilation of the Stensen's duct	blowing balloon	balloon	advice to avoid the precipitating factor (balloon-blowing)	2 days	-	2 years
Al Ohali et al. 2020 [123]	14	m	L	7 months	recurrent swelling	NR	asthma	+	+	CT	a large air sac in the left parotid gland in its superficial part extending to the duct and sparing the deep part	a habit of blowing the cheeks	abnormal habit	advice not to blow his cheeks	+	-	2 years
Fernandez et al. 2020 [124]	25	m	r	NR	sensation and noise described as a "sponge crunching"	-	adenoidectomy	+	+	US, CT	presence of air in the ductal system and within the gland	idiopathic	idiopathic	conservative measures	NR	NR	NR
Yoshida 2022 [125]	57	m	bi	1 year	recurrent swelling, pain	-	obstructive sleep apnea syndrome	+	+	CT	air within the bilateral parotid ducts and parotid parenchyma	obstructive sleep apnea syndrome, hichiriki, air flight	diseases	oral appliance	1 week	+	10 years
Goncalves et al. 2022 [126]	59	m	l	Years	NR	NR	NR	NR	+	US	hyperechoic reflexes appearing like a trail, which are termed "sunbeam effects" (major reverberation effects)	primary papilla insufficiency	idiopathic	NR	NR	NR	NR
	28	m	r	2 months	chronic parotitis	NR	chronic parotitis	NR	+	US, sialendoscopy	"sunbeam effects"	primary papilla insufficiency, chronic parotitis	idiopathic	NR	NR	NR	NR
	11	m	l	4 months	NR	NR	NR	NR	+	US	"sunbeam effects"	primary papilla insufficiency	wind instruments	NR	NR	NR	NR
	56	m	r	Years	NR	NR	duct anomaly	NR	+	US, sialendoscopy	multiple hyperechoic reflexes within the parenchyma, with strong acoustic shadowing due to the amount of reflexes, with a weak "sunbeam effects" or dirty shadow	primary papilla insufficiency, duct anomaly	idiopathic	NR	NR	NR	NR
	49	w	r	Years	chronic parotitis	NR	NR	NR	+	US, sialendoscopy	"sunbeam effects"	primary papilla insufficiency, chronic parotitis	idiopathic	NR	NR	NR	NR
	64	m	r	Months	NR	bruxism	NR	NR	+	US, sialendoscopy	"sunbeam effects"	primary papilla insufficiency	idiopathic	NR	NR	NR	NR
	20	m	r	2 years	sialectasis parotid duct system	bruxism	NR	NR	+	US	"sunbeam effects"	primary papilla insufficiency, sialectasis parotid duct system	idiopathic	NR	NR	NR	NR
	56	m	l	5–6 years	NR	NR	NR	NR	+	US, sialendoscopy	"sunbeam effects"	primary papilla insufficiency	idiopathic	NR	NR	NR	NR
	62	m	l	2 years	NR	NR	NR	NR	+	US	"sunbeam effects"	primary papilla insufficiency, blowing the nose with a closed mouth	abnormal habit	NR	NR	NR	NR
	71	m	l	Months	NR	NR	NR	NR	+	US, sialendoscopy	"sunbeam effects"	primary papilla insufficiency	idiopathic	NR	NR	NR	NR
	14	m	l	1 month	NR	NR	NR	NR	+	US, sialendoscopy	presence of multiple small, flattened, mobile, hyperechoic reflexes within the parenparenchyma, which show reverberation effects with dirty shadows and "sunbeam effects"	primary papilla insufficiency	wind instruments	NR	NR	NR	NR
	40	w	l	5 years	chronic sialectatic parotitis	NR	NR	NR	+	US, sialendoscopy	"sunbeam effects"	primary papilla insufficiency, chronic sialectatic parotitis	idiopathic	NR	NR	NR	NR
	48	w	r	1 year	NR	NR	SLE, Sjögren syndrome with MALT-lymphoma	NR	+	US, sialendoscopy	"sunbeam effects"	primary papilla insufficiency, radiotherapy	other	NR	NR	NR	NR
	41	m	r	Years	chronic parotitis	bruxism	NR	NR	+	US, sialendoscopy	"sunbeam effects"	primary papilla insufficiency, chronic parotitis	idiopathic	NR	NR	NR	NR

bi, bilateral; l, left; r, right; NR, not reported; CT, computed tomography; US, ultrasound; MRI, magnetic resonance imaging; CPAP, continuous positive airway pressure; NSAIDs, nonsteroidal anti-inflammatory drugs.
